# Congenital Pseudohorseshoe Lung Associated with Scimitar Syndrome

**DOI:** 10.5812/iranjradiol.7808

**Published:** 2012-06-30

**Authors:** Alptekin Tosun, Serife Leblebisatan

**Affiliations:** 1 Department of Radiology, Faculty of Medicine, University of Giresun, Giresun, Turkey; 2 Department of Radiology, Numune Research and Education Hospital, Adana, Turkey

**Keywords:** Congenital Abnormalities, Pulmonary Circulation, Pulmonary Atresia, Computed Tomography

## Abstract

Horseshoe lung is a congenital pulmonary malformation that is usually associated with scimitar syndrome. This malformation consists of fusion of both pulmonary lobes from the posterobasal segments. The fusion appears in the retrocardiac area, in front of the esophagus and thoracic aorta. Pleural separation of pulmonary lobes distinguishes pseudohorseshoe appearance from a true horseshoe lung. Scimitar syndrome known as hypogenetic lung syndrome is a part of the congenital pulmonary venolobar syndrome. It is a partial anomalous pulmonary venous return with pulmonary hypoplasia. Scimitar vein is an anomalous drainage vessel between the right pulmonary lobe vessels and the inferior vena cava. The appearance of the vessel resembles Turkish scimitar; therefore, the syndrome is called scimitar syndrome. We hereby report a 61-year-old woman with adult form congenital scimitar syndrome and will describe the imaging findings of pseudohorseshoe lung appearance.

## 1. Introduction

Horseshoe lung is an uncommon variant of hypogenetic lung syndrome. It is a partial fusion of the right and left pulmonary lobes from posterobasal segments. The fusion occurs in the retrocardiac area and the ventral region of the esophagus, aorta and vertebral osseous tissue. The isthmus contains right bronchial distribution and the right pulmonary artery supplies the herniated tissue. Pseudohorseshoe lung malformation is a basal segment herniation of the right pulmonary lobe. The herniated portion may be attached or may be separated by pleural membranes (1, 2). Congenital pulmonary venolobar syndrome is a constellation of different congenital anomalies of the thorax. Major components are hypogenetic lung (69%), partial anomalous pulmonary venous return (scimitar syndrome) (31%), pulmonary sequestration (24%), absence of the pulmonary artery (14%), systemic arterialization of the lung without sequestration (10%), absence or interruption of the inferior vena cava (7%) and duplication of the diaphragm (7%) (2). Acquired horseshoe appearance after the pulmonary diseases may have similar imaging findings (3). In this study, the imaging findings of congenital scimitar syndrome in an adult will be reviewed. In addition, the difference between acquired and congenital forms and the difference between horseshoe and pseudohorseshoe lung appearance will be mentioned.

## 2. Case Presentation

**Figure 1:**
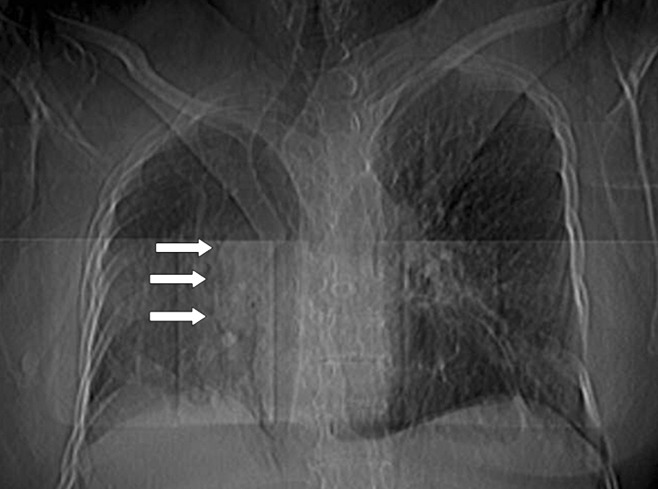
Scanogram image in a 61-year-old woman with congenital pseudohorseshoe lung associated with scimitar syndrome The right hilum is enlarged and dextroposition presents. The cardiac border of the right side is indistinct. Scimitar shaped curvilinear tubular vein is shown on the paracardiac region (arrows). Mediastinum and trachea are displaced to the right. Right pulmonary lobe hypoplasia and structural distortion is visible. Right hemidiaphragm is partially elevated.

**Figure 2:**
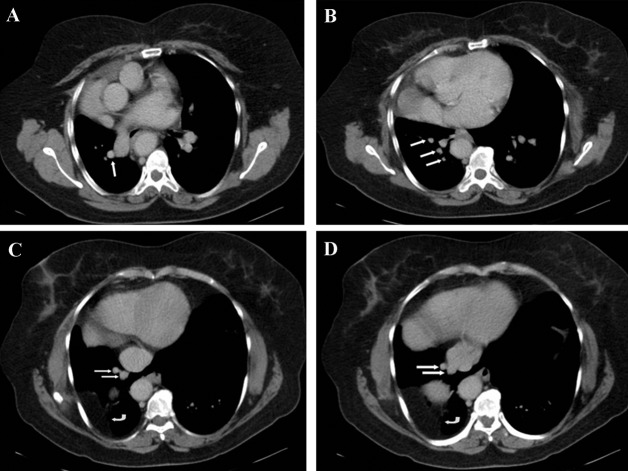
A, Contrast enhanced CT scan below the pulmonary sling in the same patient. The pulmonary vein is adjacent to the pulmonary artery (arrow). B, CT scan of the lung base. The branches of the anomalous venous structure are visible (arrows). C,D, Distal branches drained to the inferior vena cava (arrows). Abdominal fatty tissue and a part of the liver (curved arrow) are herniated to the posterobasal region of the thorax. (Bochdalek’s hernia)

**Figure 3:**
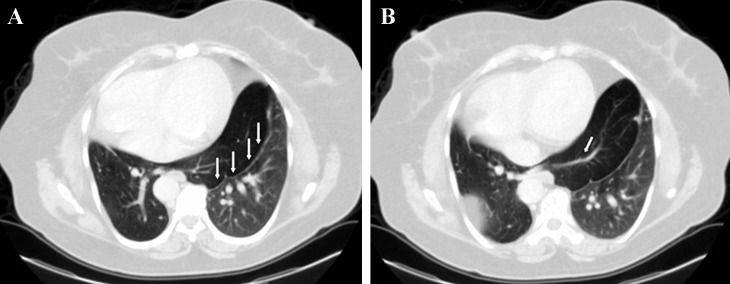
CT images in parenchyma window in the same patient A, Herniation of the right basal lobe and isthmus is visible. Herniated lobe is hyperinflated and surrounded by pleural membrane (arrows). B, Lobe bronchi contains fluid attenuation and extends to the herniated lobe (arrow). Herniated lobe is hypodense due to air remains.

A 61-year-old woman was admitted to the hospital for chest pain. She was a nonsmoker. Physical examination was nonspecific. Echocardiography revealed abnormal location of the heart on the right side. Chest roentgenogram showed dextroposition of the heart. The right pulmonary lobe was hypoplastic and the right hemithorax had structural distortion. The mediastinum was deviated to the right. The right hilum was prominent. A curvilinear scimitar like tubular lesion was observed on the right side of the cardiac border. The patient underwent thoracic CT scan. Multidetector CT examination and coronal reconstruction studies revealed a contrast filled tubular structure arising from the right pulmonary vein. The abnormal vessel had branches in the distal component. Distal branches drained to the supradiaphragmatic portion of the inferior vena cava (Figure 1, 2). The azygos vein was dilated. The right pulmonary lobe had volume loss. The upper portion was hypoplasic and the ventral component was displaced to posterior due to mediastinal shift. There was herniation of the basal segment to the left. The basal lobes, isthmus and herniated portion were hyperaerated. The lower lobe bronchi extended to the herniated portion. The herniated component was wrapped by the pleural membrane. Structural bone anomalies due to the unilateral lung hypoplasia and Bochdalek hernia on the right hemidiaphragm were observed (Figure 3). The imaging findings revealed pseudohorseshoe lung with scimitar syndrome. Symptomatic treatment was performed and the patient was asymptomatic during follow-up.

## 3. Discussion

**Figure 4:**
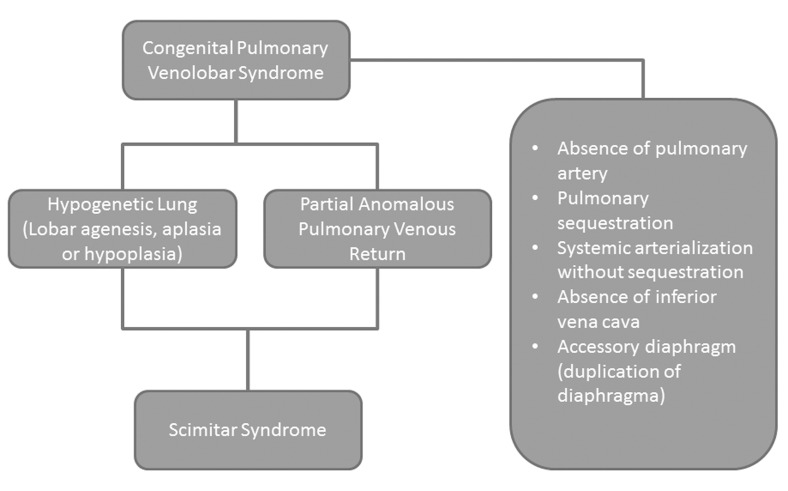
Major components of CPVS Hypogenetic lung and partial anomalous pulmonary venous return are the most common components. Scimitar syndrome displays coexistence of hypogenetic lung and PAPVR.

Congenital pulmonary venolobar syndrome (CPVS) is a group of several congenital abnormalities of the thorax. Hypogenetic lung and partial anomalous pulmonary venous return (PAPVR) is the most common anomaly of the group. Other major components are interruption of the pulmonary artery and/or inferior vena cava, pulmonary sequestration, systemic pulmonary arterializations and an accessory diaphragm. Minor components are tracheal trifurcation, eventration and partial absence of the diaphragm, phrenic cyst, horseshoe lung, superior vena cava anomalous and absence of the left pericardium (1, 2, 4) (Figure 4). Anomalous pulmonary venous drainage is an extracardiac left-to-right shunt which leads the pulmonary venous flow into the right side circulation. The disease has partial and total forms. Right-to-left shunt by a septal defect or patent ductus arteriosus is mandatory in the total form (5). PAPVR may accompany atrial septal defects and hypogenetic lung. Hypogenetic lung is a component of CPVS (4, 5, 6). Scimitar syndrome is a subtype of PAPVR in CPVS group. Hypogenetic lung may occur not only on the right side, but also in the left pulmonary lobe (5, 6, 7). Scimitar vein usually collects anomalous blood to the infradiaphragmatic portion of the inferior vena cava, although the suprahepatic vena cava, hepatic veins, portal vein, azygos vein, coronary sinus and right atrium drainage may also be seen. Left atrium drainage known meandering pulmonary vein (4, 7). Our patient has an anomalous scimitar vein which originates from the right pulmonary vein. The branches of scimitar vein drained to the supradiaphragmatic portion of the inferior vena cava. Scimitar syndrome is a rare congenital cardiopulmonary malformation and existence of horseshoe lung is extremely uncommon. This syndrome is a subtype of PAPVR in CPVS. Female involvement is higher. Familial determination is extremely rare. The syndrome is usually asymptomatic and has an incidental finding in adults; however, it is more symptomatic in early ages. Right pulmonary lobe hypoplasia exlains dextroposition of the heart and the mediastinum shift. Congenital heart disease also increases the diagnostic ratio in childhood (8). Pulmonary aplasia is complete absence of the lobe tissue and airways. The rudimental bronchus shows sudden occlusion (4). Horseshoe lung is characterized as fusion of the pulmonary lobes with an isthmus in the midline. The lobes may or may not be separated by a pleural mem- brane. The membrane is a clue of nonunion; therefore, this pseudofusion displays congenital pseudohorseshoe lung. Nonexistence of the pleural membrane is a true horseshoe lung malformation (2, 3). We believe that this terminology should only be used in congenital malformations. Many diseases such as necrotizing pneumonia cause volume loss and a destroyed lung. The nonaffected lobe may expose compensatory hypertrophy. Acquired pulmonary diseases may lead to inflammatory conditions and fibrosis in the lung tissue. This situation may cause an acquired horseshoe appearance. Pulmonary hypoplasia may exist in scimitar syndrome. Aplasia of the pulmonary artery shines a light on pulmonary lobe agenesis or hypoplasia. The hypoplastic tissue is drained by peripheral bronchial arteries of the systemic collateral circulation (4). Scimitar syndrome is characterized by hypoplasia of the right pulmonary lobe and pulmonary artery with anomalous venous connection (9, 10, 11). Diaphragmatic anomalies exist in CPVS. These anomalies include congenital weakness, eventration, accessory leaf and congenital diaphragmatic defects. Elevation of hemidiaphragm and abdominal tissue herniation may occur in scimitar syndrome (4, 8). Small posterior diaphragmatic hernia (Bochdalek hernia) appears in 6% of the population and left side involvement has dominance. Morgagni hernia is less common. The defect usually occurs in the ventromedial area of the right leaf (6). Hemivertebral anomalies may also exist in the disease (8).
